# Living Alone With Dementia Is a Neglected Source of Inequality: Findings From a Scoping Review

**DOI:** 10.1093/geroni/igaf122.884

**Published:** 2025-12-31

**Authors:** Linda Clare, Anthony Martyr, Laura Gamble, Maria Caulfield, Catherine Charlwood, Claire Hulme, Jan Oyebode, Matthew Prina

**Affiliations:** University of Exeter, Exeter, England, United Kingdom; University of Exeter, Exeter, England, United Kingdom; Newcastle University, Newcastle upon Tyne, England, United Kingdom; University of Bradford, Bradford, England, United Kingdom; University of Exeter Medical School, Exeter, England, United Kingdom; University of Exeter, Exeter, England, United Kingdom; Bradford University, Bradford, England, United Kingdom; Newcastle University, Newcastle upon Tyne, England, United Kingdom

## Abstract

The finding that 40% of community-dwelling individuals with dementia in England live alone challenges still-prevalent assumptions about availability of informal carers. We conducted a scoping review to synthesise knowledge about their characteristics and needs and how best to support them, in consultation with people with lived experience and other stakeholders. Following PRISMA-ScR guidelines, we searched for English-language publications in seven databases without date restriction. After screening, 200 articles reporting on 161 discrete studies met inclusion criteria. Findings showed that people living alone with dementia are more likely to be female and older, with pre-existing social disadvantage, significant unmet needs, and varying levels of informal support. Although no different to those living with others in dementia symptoms or general health, people living alone are subject to inequalities in relation to diagnosis, provision of formal support, financial burden and transition to institutional care. They experience greater loneliness and isolation and more challenges in everyday life. Family members and professionals are primarily concerned with balancing risk and autonomy, but family members involved at a distance receive little support. Although people living alone have more unmet needs and different needs to those living with others, surprisingly few studies explored ways of addressing these needs or improving support. We conclude that living alone with dementia is a neglected source of inequality. We offer recommendations for policy and practice in four key areas: robust estimation of numbers, increasing service responsiveness, strengthening community support, and ensuring research is inclusive and focused on practical ways of improving support.

